# Associations between a series of composite glucose-lipid indices and moderately increased albuminuria in patients with type 2 diabetes mellitus: a cross-sectional study

**DOI:** 10.3389/fendo.2026.1883785

**Published:** 2026-10-08

**Authors:** Yaru Li, Zhenyu Pan, Xinbo Zhao, Guanqi Gao, Dan Fu, Baolan Ji

**Affiliations:** 1 School of Clinical Medicine, Shandong Second Medical University, Weifang, Shandong, China; 2 Department of Endocrinology, Linyi People’s Hospital, Shandong Second Medical University, Linyi, Shandong, China; 3 Teaching and Research Section of Diagnostics, Shandong Medical College, Linyi, Shandong, China

**Keywords:** composite glucose-lipid indices, diabetic kidney disease, moderately increased albuminuria, triglyceride-glycated hemoglobin index, type 2 diabetes mellitus

## Abstract

**Purpose:**

Diabetic kidney disease (DKD) represents one of the most significant microvascular complications associated with type 2 diabetes mellitus (T2DM). Moderately increased albuminuria, defined as a urinary albumin-to-creatinine ratio (UACR) of 30–300 mg/g, is widely recognized as an early indicator of renal injury in patients with T2DM. Previous studies have confirmed the role of glucolipotoxicity in the progression of DKD. Recently, a series of composite indices originating from blood glucose and lipid levels have been proposed, covering triglyceride-glucose index (TyG), triglyceride-glucose index to high-density lipoprotein cholesterol ratio (TyG/HDL-c), triglyceride glucose (TyG)-atherogenic index of plasma (AIP) index (TyG-AIP), cholesterol-high-density lipoprotein-glucose index (CHG) and triglyceride-glycated hemoglobin index (TyH-i). This study aimed to investigate the association between the aforementioned composite glucose-lipid indices and moderately increased albuminuria in patients with T2DM.

**Methods:**

The cross-sectional analysis included 1743 patients with T2DM. The clinical and laboratory data were obtained and related indices were calculated. Patients were categorized into the moderately increased albuminuria group (UACR 30–300 mg/g) and the normal to mildly increased albuminuria group (UACR < 30 mg/g).

**Results:**

This study encompassed 342 participants with moderately increased albuminuria. The composite glucose-lipid indices levels were elevated in the moderately increased albuminuria group (all P < 0.05). Univariate analysis revealed that these indices all exhibited a positive relationship with moderately increased albuminuria (all P < 0.05). Logistic regression analysis showed that TyH-i was the only composite glucose-lipid index retained in the logistic model (OR: 1.789; 95% CI: 1.276 - 2.510). In separate adjusted models, TyH-i remained independently associated with moderately increased albuminuria (OR: 2.279; 95% CI: 1.671 - 3.110).

**Conclusion:**

Among numerous composite glucose-lipid indices, TyH-i remained independently associated with moderately increased albuminuria after adjustment. However, its discriminatory performance was limited and did not differ significantly from that of the comparator indices.

## Introduction

The worldwide concern about type 2 diabetes mellitus (T2DM) is escalating, posing deepening challenges for health systems and communities across the globe (1). Inappropriately controlled T2DM has been shown to result in manifold complications, such as vascular, neurological, and multi-organ issues (2). Among the microvascular complications of T2DM, diabetic kidney disease (DKD) is highly prevalent (3). On a global scale, end-stage renal disease (ESRD) is most frequently attributed to DKD (4). Research indicated that roughly 30-40% of diabetic patients are inclined to develop DKD (5). An albumin-to-creatinine ratio (UACR) level exceeding 30 mg/g but remaining below 300 mg/g defines moderately increased albuminuria (historically termed microalbuminuria), which is indicative of early-stage DKD (6, 7). Moreover, research has verified that moderately increased albuminuria is a predisposing factor for developing both ESRD and cardiovascular diseases (CVD) (8). Thus, early identification and prevention of moderately increased albuminuria are critical, which can arrest the initiation and worsening of DKD.

The progression of DKD is driven by glucolipotoxicity. Sustained hyperglycemia disrupts metabolic mediators such as reactive oxygen species and advanced glycation end products from an overall perspective, while it induces aberrant renal hemodynamics and glomerular hyperfiltration at the renal level (9). In addition, renal lipotoxicity, a key driver of DKD that arises from disordered lipid uptake, oxidation, efflux, and synthesis within renal cells, involves accumulated toxic lipid intermediates that disrupt mitochondria, exacerbate oxidative and endoplasmic reticulum stress, trigger inflammation and fibrosis, and eventually lead to renal function decline (10). Therefore, simple and readily available composite indices integrating blood glucose and lipid parameters may help capture the combined effects of glucolipotoxicity, whereas a single traditional glucose or lipid parameter may not fully reflect these synergistic effects.

Recent advances have enabled the development of novel composite indices that combine blood glucose and lipid parameters, thereby providing a more comprehensive assessment of glucolipotoxicity. The triglyceride-glucose index (TyG) is a classical sign of glucolipotoxicity (11), constructed by fasting plasma glucose (FPG) and triglycerides (TG) and is associated with DKD (12). Subsequently, the researchers put forward a series of derivative indices of the TyG index. The triglyceride-glucose index to high-density lipoprotein cholesterol ratio (TyG/HDL-c) has been shown to be significantly associated with diabetes in metabolic-associated fatty liver disease (13). The triglyceride glucose (TyG)-atherogenic index of plasma (AIP) index (TyG-AIP) correlates with elevated stroke risk, particularly among individuals with dysglycemia (14). Furthermore, the triglyceride-glycated hemoglobin index (TyH-i), a novel composite index analogous to the TyG index that integrates glycated hemoglobin (HbA1c) and TG, has demonstrated clinical utility in cohorts of patients with T2DM and DKD (15, 16). Moreover, the cholesterol-high-density lipoprotein-glucose index (CHG) also serves as a novel biomarker that is akin to the TyG index, demonstrates a marked correlation with DKD (17).

Currently, most studies have focused on single or a limited set of composite glucose-lipid indices, and forceful systematic evidence linking these indices to T2DM and its complications remains scarce. In addition, considering that the pathophysiological mechanisms of DKD differ between the early stage and the middle to late stages (18). Therefore, we employed a suite of complementary statistical approaches to explore the association between the composite glucose-lipid indices and moderately increased albuminuria in patients with T2DM.

## Methods

### Research methods and sample characteristics

For this study, participants aged 18 to 90 years diagnosed with T2DM were enrolled at Linyi People’s Hospital’s Department of Endocrinology during the time frame of March 2020 to February 2023. All patients were diagnosed in accordance with the 1999 World Health Organization (WHO) diagnostic criteria. The criteria listed below were used for exclusion: (1) patients suffering from different forms of diabetes; (2) individuals with UACR exceeding 300 mg/g; (3) patients diagnosed with severe liver (beyond two-fold of the normal upper threshold) or kidney involvement (eGFR < 60 mL/min/1.73m^2^); (4) patients have urinary tract infections; (5) medical records that were not complete, including patients with missing UACR data and missing data for calculating composite glucose-lipid indices. Ultimately, our study included 1743 patients with T2DM.

### General conditions and physical assessments

The participants’ baseline characteristics, including sex, smoking and drinking habits, age, duration of diabetes, height and weight were documented. Information regarding the use of sodium-glucose cotransporter 2 (SGLT2) inhibitors, glucagon-like peptide-1 (GLP-1) receptor agonists, angiotensin-converting enzyme inhibitors (ACEIs), angiotensin receptor blockers (ARBs), statins, fibrates, metformin, and insulin was collected. Following a sufficient period of rest, a standard electronic device was utilized to gather the systolic blood pressure (SBP) and diastolic blood pressure (DBP). Bioelectrical impedance analysis (HDS-2000; Omron, Kyoto, Japan) served for the assessment of the visceral fat area (VFA) and subcutaneous fat area (SFA).

### Laboratory measurements

Early morning venous samples were obtained and submitted to biochemical profiling using an automated system (Cobas c 702, Roche, Germany) after a minimum of 8 hours of sustained fasting. The total cholesterol (TC), TG, HDL-c, low-density lipoprotein cholesterol (LDL-c), aspartate aminotransferase (AST), alanine aminotransferase (ALT), gamma-glutamyl transferase (GGT), uric acid (UA), serum creatinine (Scr), hemoglobin (Hb), FPG and HbA1c (high-performance liquid chromatography) were tested. Fasting serum insulin (FINS) levels were determined via a fully automated machine (Aptio Automation, Siemens, USA). UACR was obtained through an autoanalyzer (Beckman Coulter AU5821).

### Diagnostic criteria and assessment of diabetic retinopathy

Diagnose DR in accordance with established clinical criteria (19).

### Parameter calculations

Homeostasis model assessment index (HOMA-IR) = FPG (mmol/L) * FINS (µU/mL)/22.5 (20);

TyG index = Ln [TG (mg/dL) * FPG (mg/dL)/2] (21);

TyG/HDL-c = TyG index/HDL-c (mmol/L) (22);

Atherogenic index of plasma (AIP) = log_10_ [TG (mg/dl)/HDL-c (mg/dl)] (23);

TyG-AIP =TyG * [AIP - min (AIP)]/[max (AIP) - min (AIP)] (23);

CHG index = Ln [TC (mg/dL) * FPG (mg/dL)/2 * HDL-c (mg/dL)] (24);

"TyH-i = Ln [HbA1c (%) * TG (mg/dL)/2] (15);

Body mass index (BMI) = weight (kg)/height (m)^2^ (25);

eGFR = 175 * Scr (mg/dL) ^- 1.234^ * age ^- 0.179^ × (0.79, if female) (26).

### Statistical methods

SPSS 25.0 (SPSS Inc., Chicago, USA), R software (version 4.6.1, R Foundation for Statistical Computing, Vienna, Austria), and SPSSAU (https://spssau.com) were utilized to perform the data analysis. Candidate variables were first selected based on clinical knowledge and prior literature. Non-normally distributed variables were presented as median and interquartile range and compared using the Mann-Whitney U test, normally distributed variables were presented as mean ± SD and compared using independent samples t-tests. Categorical variables were presented as percentages and compared using the chi-square test. Univariate analysis was then performed on these candidate variables, and variables with P < 0.05 were entered into binary stepwise forward logistic regression for further selection. The Hosmer-Lemeshow test was employed to assess whether the model exhibits a lack of fit.

Correlation coefficients and variance inflation factors (VIF) were used to examine multicollinearity. Separate multivariable models, each including one indicator from the multicollinearity set on top of common confounders, were constructed for sensitivity analysis. The reference model for Net Reclassification Improvement (NRI) and Integrated Discrimination Improvement (IDI) included only common confounders. Continuous NRI was employed. Akaike Information Criterion (AIC) and Bayesian Information Criterion (BIC) were computed for each model for descriptive purposes. Restricted cubic spline (RCS) analysis was used as an exploratory method to examine the potential nonlinear association between TyH-i and moderately increased albuminuria. ROC curves and DeLong tests were used to assess and compare discriminatory performance. LASSO regression was used as an exploratory penalized variable-selection approach. Two-tailed P < 0.05 was considered statistically significant.

## Results

### Baseline clinical and biochemical characteristics

**Table 1** displays the clinical and biochemical characteristics of participants. Compared with the normal to mildly increased albuminuria group, the moderately increased albuminuria group had elevated levels of age, duration of diabetes, BMI, VFA, SBP, DBP, TC, TG, FPG, FINS, HbA1c, HOMA-IR, GGT, UA, the percentage of ACEIs or ARBs, the percentage of insulin, UACR, Scr, TyG index, TyG/HDL-C, TyG-AIP, CHG index, TyH-i, and the percentage of DR (all P < 0.05). Furthermore, HDL-c, eGFR, Hb, and the percentage of metformin were significantly lower in the moderately increased albuminuria group (all P < 0.05). However, sex, the percentage of smoking and drinking, SFA, LDL-c, AST, ALT, the percentage of SGLT2 inhibitors, the percentage of GLP-1 receptor agonists, the percentage of statins, and the percentage of fibrates did not differ significantly between the two groups (all P > 0.05).

**Table 1:** Clinical and biochemical characteristics by presence of moderately increased albuminuria.

Variables	AII	Normal to mildly increased albuminuria group	Moderately increased albuminuria group	P
Numbers	1743	1401	342	
Sex (Females, n, %)	896 (51.4%)	720 (51.4%)	176 (51.5%)	0.981
Smoking (%)	337 (19.3%)	267 (19.1%)	70 (20.5%)	0.554
Drinking (%)	323 (18.5%)	258 (18.4%)	65 (19.0%)	0.801
Age (years)	57.3 ± 12.4	56.9 ± 12.2	58.8 ± 13.4	0.009
Duration of diabetes (years)	6.0 (1.5 - 10.0)	6.0 (1.0 - 10.0)	8.0 (3.0 - 14.0)	< 0.001
BMI (kg/m^2^)	25.66 ± 3.54	25.56 ± 3.40	26.09 ± 4.03	0.013
VFA (cm^2^)	91.00 (68.00 - 120.00)	90.00 (67.00 - 119.00)	98.00 (71.00 - 127.00)	0.005
SFA (cm^2^)	184.00 (142.50 - 229.00)	182.00 (142.00 - 227.75)	191.00 (143.50 - 238.50)	0.085
SBP (mmHg)	129.7 ± 18.3	128.5 ± 17.3	134.5 ± 21.1	< 0.001
DBP (mmHg)	80.8 ± 11.4	80.4 ± 10.7	82.3 ± 13.9	0.009
TC (mmol/L)	4.75 ± 1.16	4.72 ± 1.14	4.87 ± 1.24	0.036
TG (mmol/L)	1.37 (0.97 - 2.03)	1.35 (0.95 - 1.97)	1.50 (1.02 - 2.22)	0.004
HDL-c (mmol/L)	1.16 ± 0.32	1.17 ± 0.32	1.13 ± 0.31	0.038
LDL-c (mmol/L)	3.03 ± 0.98	3.02 ± 0.96	3.09 ± 1.06	0.229
FPG (mmol/L)	8.86 ± 3.54	8.71 ± 3.45	9.45 ± 3.84	0.001
FINS (µU/mL)	15.96 (9.43 - 22.11)	15.43 (8.78 - 21.48)	18.14 (11.86 - 24.48)	< 0.001
HbA1c (%)	9.18 ± 2.02	9.08 ± 2.00	9.61 ± 2.07	< 0.001
HOMA-IR	5.83 (3.07 - 9.35)	5.42 (2.90 - 8.96)	7.30 (4.47 - 11.20)	< 0.001
AST (U/L)	17.20 (14.00 - 21.70)	17.30 (14.00 - 21.70)	17.10 (14.00 - 21.93)	0.857
ALT (U/L)	18.20 (13.60 - 25.70)	18.20 (13.60 - 25.80)	18.10 (13.15 - 25.25)	0.556
GGT (U/L)	21.00 (16.00 - 31.08)	21.00 (15.00 - 30.88)	23.70 (16.38 - 35.25)	0.005
UA (μmol/L)	280.61 ± 86.12	276.11 ± 82.88	299.06 ± 96.21	< 0.001
UACR (mg/g)	8.90 (5.10 - 22.80)	7.10 (4.60 - 12.15)	67.60 (43.20 - 121.28)	< 0.001
Scr (µmol/L)	61.74 ± 13.75	60.83 ± 12.94	65.46 ± 16.14	< 0.001
eGFR (mL/min/1.73 m^2^)	124.56 ± 29.98	126.40 ± 29.36	116.99 ± 31.32	< 0.001
Hb (g/L)	143.07 ± 17.63	143.88 ± 16.66	139.79 ± 20.83	< 0.001
SGLT2 inhibitors, n (%)	92 (5.3%)	71 (5.1%)	21 (6.1%)	0.421
GLP-1 receptor agonists, n (%)	381 (21.9%)	304 (21.7%)	77 (22.5%)	0.729
ACEIs or ARBs, n (%)	302 (17.3%)	214 (15.3%)	88 (25.7%)	< 0.001
Statins, n (%)	1253 (71.9%)	993 (70.9%)	260 (76.0%)	0.050
Fibrates, n (%)	65 (3.7%)	48 (3.4%)	17 (5.0%)	0.174
Metformin, n (%)	1140 (65.4%)	944 (67.4%)	196 (57.3%)	0.001
Insulin, n (%)	1405 (80.6%)	1104 (78.8%)	301 (88.0%)	< 0.001
TyG index	9.15 ± 0.74	9.12 ± 0.73	9.29 ± 0.79	< 0.001
TyG/HDL-c	8.45 ± 2.51	8.35 ± 2.46	8.85 ± 2.67	0.001
TyG-AIP	4.59 ± 1.74	4.52 ± 1.71	4.89 ± 1.83	< 0.001
CHG index	5.72 ± 0.53	5.69 ± 0.51	5.83 ± 0.58	< 0.001
TyH-i	6.34 ± 0.62	6.31 ± 0.61	6.47 ± 0.65	< 0.001
DR	626 (35.9%)	462 (33.0%)	164 (48.0%)	< 0.001

The distribution of the variables was normally approximated by a mean ± standard deviation, and comparisons between the two groups were conducted using an independent samples t-test. Variables with abnormally distributed data were expressed as the median (25th percentile to 75th percentile), and comparisons between the two groups were conducted using the Mann–Whitney U-test. Categorical variables were expressed as percentages and subjected to a chi-square test for comparison. Statistical differences were defined by a P value less than 0.05 (two-tailed).

Normal to mildly increased albuminuria group, UACR< 30 mg/g; Moderately increased albuminuria group, UACR 30-300 mg/g; BMI, body mass index; VFA, visceral fat area; SFA, subcutaneous fat area; SBP, systolic blood pressure; DBP, diastolic blood pressure; TC, total cholesterol; TG, triglyceride; HDL-c, high-density lipoprotein cholesterol; LDL-c, low-density lipoprotein cholesterol; FPG, fasting plasma glucose; FINS, fasting serum insulin; HbA1c, glycosylated hemoglobin; HOMA-IR, homeostatic model assessment of insulin resistance;

AST, aspartate aminotransferase; ALT, alanine aminotransferase; GGT, gamma-glutamyl transferase; UA, uric acid; UACR, urinary albumin to creatinine ratio; Scr, serum creatinine; eGFR, estimated glomerular filtration rate; Hb, hemoglobin; S GLT2 inhibitors, sodium glucose co-transporter 2 inhibitors; GLP-1 receptor agonists, glucagon-like peptide-1 receptor agonists; ACEI, angiotensin-converting enzyme inhibitor; ARB, angiotensin II receptor blocker; TyG index, triglyceride–glucose index; TyG/HDL-c, triglyceride-glucose index to high-density lipoprotein cholesterol ratio; TyG-AIP, triglyceride glucose (TyG)-atherogenic index of plasma (AIP) index; CHG index, cholesterol-high-density lipoprotein-glucose index; TyH-i, triglyceride-glycated hemoglobin index; DR, diabetic retinopathy.

### Univariate analysis

**Table 2** presents the univariate analysis of factors associated with moderately increased albuminuria. Age, duration of diabetes, VFA, SBP, TC, TG, FPG, FINS, HbA1c, HOMA-IR, GGT, UA, ACEIs or ARBs, insulin, TyG index, TyG/HDL-c, TyG-AIP, CHG index, TyH-i, and DR were positively correlated with moderately increased albuminuria (all P < 0.05). HDL-c, eGFR, Hb, and metformin were negatively correlated with moderately increased albuminuria (all P < 0.05). Notably, no significant correlations with moderately increased albuminuria were shown for sex, smoking, drinking, BMI, SFA, DBP, LDL-c, AST, ALT, SGLT2 inhibitors, GLP-1 receptor agonists, statins, and fibrates (all P > 0.05). As shown in **Supplementary Appendix Figure 1**, a range of -0.5 to 1.0 was observed for the Spearman correlations among all variables. Correlation values spanned from a moderate negative association (-0.5) to a perfect positive association (1.0).

**Table 2:** Univariate correlation analysis for moderately increased albuminuria.

Variables	Correlation coefficient	P
Sex	-0.001	0.981
Smoking	0.014	0.554
Drinking	0.006	0.801
Age	0.058	0.016
Duration of diabetes	0.113	<0.001
BMI	0.040	0.092
VFA	0.069	0.005
SFA	0.043	0.085
SBP	0.115	<0.001
DBP	0.047	0.050
TC	0.049	0.041
TG	0.070	0.004
HDL-c	-0.058	0.015
LDL-c	0.025	0.298
FPG	0.086	<0.001
FINS	0.118	<0.001
HbA1c	0.106	<0.001
HOMA-IR	0.156	<0.001
AST	0.004	0.857
ALT	-0.014	0.556
GGT	0.067	0.005
UA	0.102	<0.001
eGFR	-0.130	<0.001
Hb	-0.082	0.001
SGLT2 inhibitors	0.019	0.421
GLP-1 receptor agonists	0.008	0.729
ACEIs or ARBs	0.110	<0.001
Statins	0.047	0.050
Fibrates	0.033	0.174
Metformin	-0.083	0.001
Insulin	0.095	<0.001
TyG index	0.093	<0.001
TyG/HDL-c	0.076	0.002
TyG-AIP	0.080	0.001
CHG index	0.100	<0.001
TyH-i	0.099	<0.001
DR	0.124	<0.001

To identify the factors associated with moderately increased albuminuria, a univariate correlation analysis was conducted.

Moderately increased albuminuria, UACR 30-300 mg/g; BMI, body mass index; VFA, visceral fat area; SFA, subcutaneous fat area; SBP, systolic blood pressure; DBP, diastolic blood pressure; TC, total cholesterol; TG, triglyceride; HDL-c, high-density lipoprotein cholesterol; LDL-c, low-density lipoprotein cholesterol; FPG, fasting plasma glucose; FINS, fasting serum insulin; HbA1c, glycosylated hemoglobin; HOMA-IR, homeostatic model assessment of insulin resistance; AST, aspartate aminotransferase; ALT, alanine aminotransferase; GGT, gamma-glutamyl transferase; UA, uric acid; eGFR, estimated glomerular filtration rate; Hb, hemoglobin; SGLT2 inhibitors, sodium glucose co-transporter 2 inhibitors; GLP-1 receptor agonists, glucagon-like peptide-1 receptor agonists; ACEI, angiotensin-converting enzyme inhibitor; ARB, angiotensin II receptor blocker; TyG index, triglyceride–glucose index; TyG/HDL-c, triglyceride-glucose index to high-density lipoprotein cholesterol ratio; TyG-AIP, triglyceride glucose (TyG)-atherogenic index of plasma (AIP) index; CHG index, cholesterol-high-density lipoprotein-glucose index; TyH-i, triglyceride-glycated hemoglobin index; DR, diabetic retinopathy.

### Multivariate analysis

#### Multivariate logistic regression analysis

In the present study, moderately increased albuminuria was recognized as the dependent variable. Variables with statistical significance in the univariate correlation analysis were included in the regression model. Our study adjusted for age, duration of diabetes, VFA, SBP, TC, TG, HDL-c, FPG, FINS, HbA1c, HOMA-IR, GGT, UA, eGFR, Hb, ACEIs or ARBs, insulin, metformin, TyG, TyG/HDL-c, TyG-AIP, CHG, TyH-i and DR. **Table 3** presents the outcomes of binary stepwise forward logistic regression analysis, which revealed the independent correlation of moderately increased albuminuria. Results showed that TyH-i (OR: 1.789, 95% CI: 1.276 - 2.510), DR (OR: 1.872, 95% CI: 1.289 - 2.721), ACEIs or ARBs (OR: 1.791, 95% CI: 1.149 - 2.792), HbA1c (OR: 1.237, 95% CI: 1.112 - 1.376), duration of diabetes (OR: 1.042, 95% CI: 1.015 - 1.069), SBP (OR: 1.015, 95% CI: 1.005 - 1.026), eGFR (OR: 0.989, 95% CI: 0.983 - 0.996), Hb (OR: 0.987, 95% CI: 0.976 - 0.998) and metformin (OR: 0.590, 95% CI: 0.403 - 0.863) were associated with moderately increased albuminuria. **Figure 1** provides a forest plot depicting the independent association of moderately increased albuminuria identified through binary stepwise forward logistic regression analysis. The Hosmer-Lemeshow test result showed that P equals 0.411.

**Table 3:** The independent variables for moderately increased albuminuria.

Variables	P	OR	95.0% CI for OR
DR	0.001	1.872	1.289 - 2.721
ACEIs or ARBs	0.010	1.791	1.149 - 2.792
TyH-i	0.001	1.789	1.276 - 2.510
HbA1c	<0.001	1.237	1.112 - 1.376
Duration of diabetes	0.002	1.042	1.015 - 1.069
SBP	0.005	1.015	1.005 - 1.026
eGFR	0.002	0.989	0.983 - 0.996
Hb	0.018	0.987	0.976 - 0.998
Metformin	0.007	0.590	0.403 - 0.863

The independent variables for moderately increased albuminuria were evaluated using a logistic regression analysis.

DR, diabetic retinopathy; ACEI, angiotensin-converting enzyme inhibitor; ARB, angiotensin II receptor blocker; TyH-i, triglyceride-glycated hemoglobin index; HbA1c, glycosylated hemoglobin; SBP, systolic blood pressure; eGFR, estimated glomerular filtration rate; Hb, hemoglobin.

**Figure 1:**
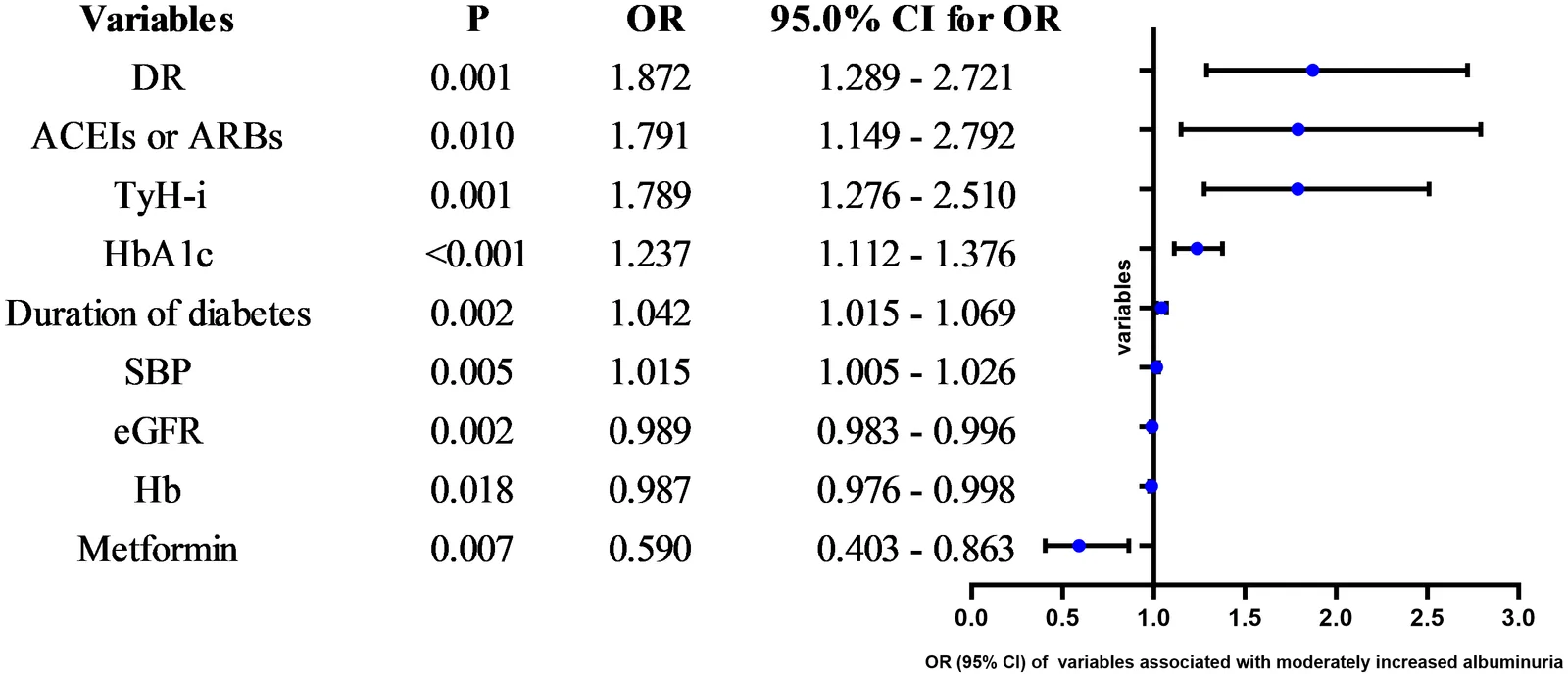
Forest plot of factors independently associated with moderately increased albuminuria. The independent variables for moderately increased albuminuria were evaluated using a logistic regression analysis. The blue solid dots represent the OR of each variable for moderately increased albuminuria and the horizontal black lines correspond to the 95% CI of OR values. The vertical line at X = 1.0 is the line of no effect. DR, diabetic retinopathy; ACEI, angiotensin-converting enzyme inhibitor; ARB, angiotensin II receptor blocker; TyH-i, triglyceride-glycated hemoglobin index; HbA1c, glycosylated hemoglobin; SBP, systolic blood pressure; eGFR, estimated glomerular filtration rate; Hb, hemoglobin.

#### Model performance metrics and RCS analysis

Due to the similarities among the components of the indices, our study examined multicollinearity by assessing the correlation coefficients and VIF among these variables. The results revealed that there existed a covariance problem between FPG, HbA1c, TG, TyG index, TyG/HDL-c, TyG-AIP, CHG index, and TyH-i (as illustrated in **Supplementary Appendix Tables 1**, **2**). Therefore, we constructed separate multivariable models, each including only one index alongside the relevant clinical covariates. TyH-i remained independently associated with moderately increased albuminuria (OR 2.279, 95% CI 1.671 - 3.110; **Table 4**). NRI and IDI were calculated as exploratory reclassification metrics (as shown in **Table 4**), and the corresponding P values and 95% CI were provided in **Supplementary Appendix Tables 5**, **6**. AIC and BIC values for each model were also summarized in **Table 4**. The AUC values of the composite glucose-lipid indices for identifying moderately increased albuminuria were all below 0.60 (**Supplementary Appendix Table 3**; **Supplementary Appendix Figure 3**), and DeLong tests showed no statistically significant differences between indices (all P > 0.05; **Supplementary Appendix Table 4**). In the RCS analysis (as shown in **Figure 2**), 4 knots were used, and the reference value was set at the median of TyH-i (6.310). The overall model fit test was significant (P < 0.001), whereas the nonlinearity test was not significant (P = 0.109; **Table 5**).

**Figure 2:**
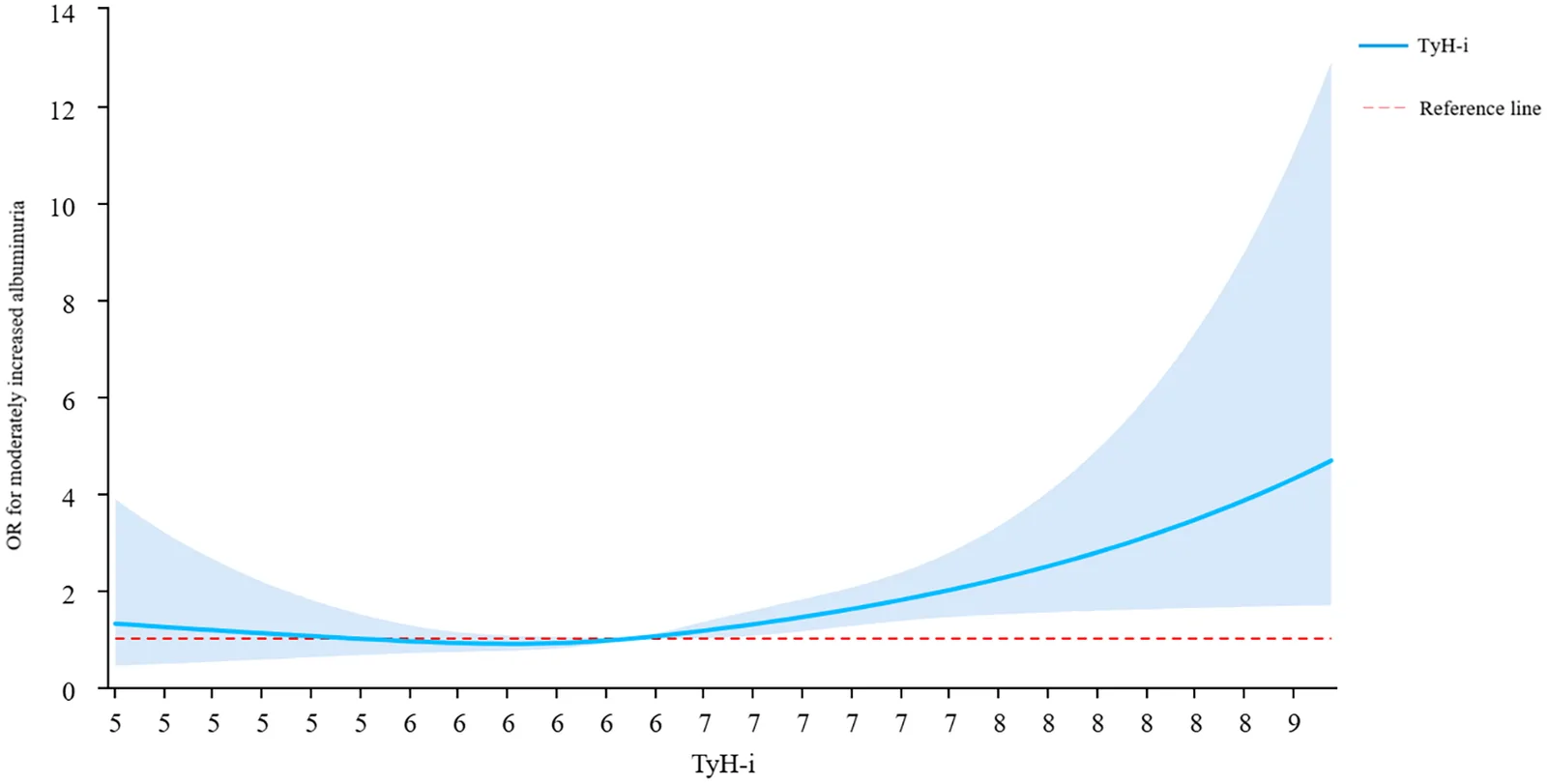
RCS analysis of TyH-i with moderately increased albuminuria.The likelihood ratio test was used to assess the overall nonlinear association between TyH-i and moderately increased albuminuria. Moderately increased albuminuria, UACR 30-300 mg/g; TyH-i, triglyceride-glycated hemoglobin index.

**Table 4:** Sensitivity analysis in different variables for moderately increased albuminuria.

Models	Variables	OR	95% CI for OR	AIC	BIC	NRI	IDI
Model 1	HbA1c	1.346	1.217-1.488	773.093	826.232	32.503%*	0.035*
Model 2	TyH-i	2.279	1.671-3.110	784.559	828.037	25.762% *	0.015*
Model 3	CHG index	2.578	1.784-3.725	785.764	829.242	22.429%*	0.017*
Model 4	TyG index	1.919	1.481-2.487	788.567	827.214	11.580%	0.009
Model 5	FPG	1.089	1.036-1.144	790.210	843.349	24.516%*	0.017*
Model 6	TyG-AIP	1.239	1.118-1.372	793.173	836.651	13.223%*	0.010*
Model 7	TyG/HDL-c	1.124	1.050-1.204	797.986	841.464	8.095%	0.003
Model 8	TG	1.302	1.126-1.506	798.284	836.931	-5.010%	-0.003

Model: Common confounding variables: age, duration of diabetes, VFA, SBP, TC, HDL-c, FINS, GGT, UA, eGFR, HOMA-IR, Hb, ACEIs or ARBs, Metformin, Insulin, DR.

Model 1: Common confounding variables + HbA1c.

Model 2: Common confounding variables + TyH-i.

Model 3: Common confounding variables + CHG index.

Model 4: Common confounding variables + TyG index.

Model 5: Common confounding variables + FPG.

Model 6: Common confounding variables + TyG-AIP.

Model 7: Common confounding variables + TyG/HDL-c.

Model 8: Common confounding variables + TG.

Models 1–8 incorporated only one indicator from the multicollinearity set on top of common confounding variables. The reference model for NRI and IDI includes only common confounding variables. Continuous NRI was employed. * means that the NRI/IDI metrics exhibited statistically significant differences when compared with the reference model.

TG, triglyceride; FPG, fasting plasma glucose; HbA1c, glycosylated hemoglobin; TyG index, triglyceride–glucose index; TyG/HDL-c, triglyceride-glucose index to high-density lipoprotein cholesterol ratio; TyG-AIP, triglyceride glucose (TyG)-atherogenic index of plasma (AIP) index; CHG index, cholesterol-high-density lipoprotein-glucose index; TyH-i, triglyceride-glycated hemoglobin index; AIC, Akaike information criterion; BIC, Bayesian information criterion; NRI, Net reclassification improvement; IDI, Integrated Discrimination Improvement. * Indicates P < 0.05.

**Table 5:** Overall nonlinear relationship test of the model for moderately increased albuminuria.

Model	-2 Log-likelihood	χ²	df	P
Intercept-only model	1545.125			
Final model	1540.700	4.426	2	0.109

The likelihood ratio test was used to assess the overall nonlinear association between TyH-i and the moderately increased albuminuria. P-value ≥ 0.05 indicates no statistically significant evidence for nonlinearity, nonlinearity cannot be entirely ruled out.

Moderately increased albuminuria: UACR 30-300 mg/g; TyH-i, triglyceride-glycated hemoglobin index; df, degrees of freedom.

#### Exploratory LASSO regression

In LASSO regression analysis, ten-fold cross-validation was adopted to determine the optimal penalty parameter λ (lambda.1se = 0.02452612). Eleven variables with non-zero coefficients were selected: duration of diabetes, SBP, HbA1c, UA, eGFR, Hb, ACEIs or ARBs, metformin, CHG index, TyH-i, and DR. **Supplementary Appendix Figure 2A** shows the LASSO coefficient-profile plot of all patients, regression coefficients of most variables gradually shrank to zero with increasing penalty parameter lambda. **Supplementary Appendix Figure 2B** shows the 10-fold cross-validation curve used to select the penalty parameter λ.

## Discussion

Using a cross-sectional design, we explored the association between numerous composite glucose-lipid indices and moderately increased albuminuria. Univariate correlation analysis revealed a significant connection between these indices and moderately increased albuminuria in patients with T2DM. TyH-i was independently associated with moderately increased albuminuria following adjustment. Nevertheless, its discriminatory performance was limited, with no significant difference relative to other comparator indices.

A previous study confirmed that, after adjusting for confounding factors such as age, gender, and BMI, the results of logistic regression indicated that the TyG index remained significantly associated with moderately increased albuminuria (27). In this study, we observed that the TyG index was significantly elevated in the moderately increased albuminuria group but did not enter the final logistic regression model. Moreover, a study reported a statistically outstanding association between the CHG index and DKD (17). However, we did not find a significant association between the CHG index and moderately increased albuminuria. Additionally, no published studies have analyzed the association between the TyG/HDL-c and TyG-AIP and moderately increased albuminuria. Similarly, we also found that these two indices were significantly elevated in the moderately increased albuminuria group, yet they did not enter the regression model in the end.

Notably, logistic regression analysis identified only TyH-i entering the logistic model among composite glucose-lipid indices (OR = 1.789, 95% CI = 1.276 - 2.510). The Hosmer-Lemeshow test yielded P = 0.411. This result indicated that there was insufficient evidence of lack of fit. In the separate model adjustment, TyH-i remained independently associated with moderately increased albuminuria (OR 2.279; 95% CI 1.671 - 3.110). Recently, Ma et al. analyzed the correlation between TyH-i and DKD by enrolling study participants with varying degrees of albuminuria, their findings indicated that TyH-i exhibited a stronger correlation with DKD than the TyG index (16). This discovery further supports our conclusion. And in our study, the OR value of TyH-i exhibited a discrepancy between the full variables model and the separate model. This discrepancy may be attributed to the multicollinearity among indices. In the full regression model incorporating all candidate variables, TyH-i was introduced into the model simultaneously with HbA1c, TG, FPG, and other composite glucose-lipid indices, leading to variance overlap among variables, which may result in the attenuation of the effect estimate of TyH-i. By contrast, each separate model incorporated only one index from the multicollinearity set on top of common confounders, which avoided the effect competition caused by multicollinearity, and the obtained effect estimates could better reflect their independent associations. The direction of association and statistical significance remained consistent across different models, indicating that the observed association possessed a certain degree of robustness. In addition, the RCS analysis did not identify a statistically significant nonlinear association (P = 0.109).

Nonetheless, the mechanism linking TyH-i and moderately increased albuminuria remains unclear. It may encompass the following aspects. HbA1c was a critical component of TyH-i, and HbA1c could reflect the historical blood glucose control level and glycemic variability. Although FPG also served as a reliable indicator of blood glucose control (28), evidence from studies indicated a stronger association between HbA1c and DKD than FPG (29). Multiple studies demonstrated that even after subsequent improvement in glycemic levels, prior poor glycemic control could induce a sustained effect on the risk of diabetic complications known as the metabolic memory effect (30, 31), and this phenomenon, together with glycemic variability (32), triggered persistent oxidative stress, chronic inflammation, and epigenetic reprogramming to promote progressive kidney injury. HbA1c was a reliable indicator of metabolic memory and glycemic fluctuations (33). In addition, elevated TG levels represented a significant risk factor for the development of DKD (34). Emerging evidence indicated that dyslipidemia in early-stage DKD was characterized by elevated TG levels (35). This may be mediated by TG accumulation, which activated monocytes to produce inflammatory mediators, transforming growth factor beta and reactive oxygen species, leading to renal cell injury (36). However, based on the current data, we were unable to distinguish whether the relationship between TyH-i and moderately increased albuminuria was derived from the mathematical structure of the index or from a genuine biological effect.

Additionally, logistic regression analysis identified several other factors associated with moderately increased albuminuria. Undoubtedly, prolonged diabetes duration increased the risk of moderately increased albuminuria (37). As the first clinical expression of DKD, moderately increased albuminuria is also known to anticipate the onset of DR (38). HbA1c and SBP were directly positively correlated with moderately increased albuminuria, which aligns with the previous study, this finding points to a potential involvement of metabolic disturbances in the progression of moderately increased albuminuria (7). A potential explanation for the observed negative correlation between Hb and moderately increased albuminuria may be attributable to impaired kidney function affecting erythropoietin production (39). According to the guidelines, patients with high risk factors for renal injury are more likely to be prescribed ACEIs or ARBs for treatment (40). The connection between metformin and moderately increased albuminuria may be attributed to metformin effectively modulating blood glucose concentrations without promoting weight gain, thereby establishing its status as a guideline endorsed first line pharmacologic intervention for T2DM (41).

However, the AUC indicated that the predictive performance of the composite glucose-lipid indices for moderately increased albuminuria was limited. The DeLong test further revealed no statistically significant difference in discriminatory accuracy among these indices. These results demonstrated that a statistically significant independent association may not guarantee satisfactory discriminatory capacity for disease prediction (42). In addition, the LASSO regression was used as an exploratory penalized variable-selection approach. Although TyH-i remained independently associated with moderately increased albuminuria in multivariable models, this observational finding required further external validation.

Further, the retrospective, single center research design may introduce selection bias, and residual confounding factors such as lifestyle and physical exercise may have an impact on the results. At the same time, constrained by the inherent structural limitations of composite indices, these indices were retrospectively derived from metabolic parameters based on routine clinical test results, and no clinical consensus has yet been established regarding their calculation methodologies. Another, based on cross-sectional studies, reverse causality cannot be fully excluded, as incipient renal injury indicated by moderately increased albuminuria may disturb composite glucose-lipid indices homeostasis and further elevate composite indices. In this study, moderately increased albuminuria was defined as a single UACR of 30–300 mg/g, and persistent albuminuria could not be confirmed. Intra-individual variability in UACR may result in outcome misclassification. Such misclassification could distort the observed associations, reduce the accuracy of risk stratification, and introduce bias in effect estimation, thereby compromising the interpretability and reliability of the results. Therefore, although these composite glucose-lipid indices showed an independent correlation with moderately increased albuminuria, they were not suitable for standalone clinical diagnosis.

### Limitations

Several limitations should be acknowledged. Primarily, defining moderately increased albuminuria based on a single UACR measurement was a major limitation of this study. A single measurement may misclassify transient albuminuria and bias risk stratification. Future studies should adopt repeated UACR measurements to mitigate misclassification. Second, owing to the cross-sectional design, causal relationships between the composite glucose-lipid indices and moderately increased albuminuria could not be established, and cut-off values for predictive purposes could not be derived. Third, variable selection in this study was exploratory without an a priori statistical analysis plan. Relying solely on univariate correlation analysis for variable selection may compromise the precision of effect estimation. Fourth, findings from some analyses require careful interpretation. The NRI and IDI were exploratory and not based on formal model comparisons. AIC and BIC reflected goodness-of-fit rather than discriminative ability. LASSO was used solely for variable screening, not model validation. RCS analysis revealed no significant nonlinearity and was reported descriptively. Calibration was assessed exclusively via the Hosmer-Lemeshow test, a non-significant P value from this test did not confirm adequate calibration. Future studies should prospectively define analytical protocols, enhance calibration assessment, incorporate bootstrapped internal validation, and standardize exploratory methods to robustly evaluate the TyH-i’s predictive performance. In addition, analyses were restricted to participants with normal-to-mild and moderately increased albuminuria. Future multicenter large-scale studies are needed to explore the generalizability of these findings to the wider DKD population and conduct subgroup analyses, such as gender, duration of diabetes, age, and obesity status.

## Conclusion

In conclusion, among various composite glucose-lipid indices, TyH-i remained independently associated with moderately increased albuminuria after adjustment. However, its discriminatory performance was limited and did not differ significantly from that of the comparator indices. Future multicenter, longitudinal or interventional studies are needed to rigorously validate the association between TyH-i and moderately increased albuminuria.

## Data Availability

The raw data supporting the conclusions of this article will be made available by the authors, without undue reservation.
