# Supplementary material for: Associations between a series of composite glucose-lipid indices and moderately increased albuminuria in patients with type 2 diabetes mellitus: a cross-sectional study
Source: Front Endocrinol (Lausanne). 2026 Oct 8;17:1883785. doi: 10.3389/fendo.2026.1883785 (PMC13686917; doi:10.3389/fendo.2026.1883785)
Supplement: Supplementary file 1 [file SupplementaryFile1.docx]

**Appendix Table 1︱**Pearson correlation analysis among composite glucose-lipid indices.

| **Variables** |  | **TyH-i** | **TyG index** | **TyG/HDL-c** | **TyG-AIP** | **CHG index** | **TG** | **HbA1c** | **FPG** | **HOMA-IR** |
| --- | --- | --- | --- | --- | --- | --- | --- | --- | --- | --- |
| TyH-i | Pearson correlation | 1.000 | 0.909 | 0.596 | 0.913 | 0.669 | 0.854 | 0.450 | 0.391 | 0.283 |
| TyG index | Pearson correlation | 0.909 | 1.000 | 0.601 | 0.889 | 0.821 | 0.797 | 0.329 | 0.646 | 0.361 |
| TyG/HDL-c | Pearson correlation | 0.596 | 0.601 | 1.000 | 0.799 | 0.582 | 0.584 | 0.147 | 0.281 | 0.251 |
| TyG-AIP | Pearson correlation | 0.913 | 0.889 | 0.799 | 1.000 | 0.645 | 0.907 | 0.153 | 0.319 | 0.245 |
| CHG index | Pearson correlation | 0.669 | 0.821 | 0.582 | 0.645 | 1.000 | 0.478 | 0.493 | 0.793 | 0.402 |
| TG | Pearson correlation | 0.854 | 0.797 | 0.584 | 0.907 | 0.478 | 1.000 | 0.081 | 0.197 | 0.160 |
| HbA1c | Pearson correlation | 0.450 | 0.329 | 0.147 | 0.153 | 0.493 | 0.081 | 1.000 | 0.502 | 0.327 |
| FPG | Pearson correlation | 0.391 | 0.646 | 0.281 | 0.319 | 0.793 | 0.197 | 0.502 | 1.000 | 0.493 |
| HOMA-IR | Pearson correlation | 0.283 | 0.361 | 0.251 | 0.245 | 0.402 | 0.160 | 0.327 | 0.493 | 1.000 |

**Note**: Correlation coefficients between TyH-i and different indices were determined by pearson correlation analysis.

**Abbreviations**: TyH-i, triglyceride-glycated hemoglobin index; TyG index, triglyceride–glucose index; TyG/HDL-c, triglyceride-glucose index to high-density lipoprotein cholesterol ratio; TyG-AIP, triglyceride glucose (TyG)-atherogenic index of plasma (AIP) index; CHG index, cholesterol-high-density lipoprotein-glucose index; TG, triglyceride; FPG, fasting plasma glucose; HbA1c, glycosylated hemoglobin; HOMA-IR, homeostatic model assessment of insulin resistance.

**Appendix table 2︱**Diagnosis of multicollinearity among indices.

| **Variables** | **VIF** |
| --- | --- |
| TyG-AIP | 224.084 |
| TyH-i | 147.222 |
| TyG index | 71.812 |
| TyG/HDL-c | 26.608 |
| HbA1c | 20.321 |
| FPG | 14.143 |
| TG | 10.708 |
| CHG index | 7.547 |
| HOMA-IR | 1.390 |

**Note**: Variance Inflation Factor (VIF) is used to diagnose multicollinearity among indices.

**Abbreviations**: TyH-i, triglyceride-glycated hemoglobin index; TyG index, triglyceride–glucose index; TyG/HDL-c, triglyceride-glucose index to high-density lipoprotein cholesterol ratio; TyG-AIP, triglyceride glucose (TyG)-atherogenic index of plasma (AIP) index; CHG index, cholesterol-high-density lipoprotein-glucose index; TG, triglyceride; FPG, fasting plasma glucose; HbA1c, glycosylated hemoglobin; HOMA-IR, homeostatic model assessment of insulin resistance.

**Appendix Table 3 |** ROC curve diagnostic parameters of different indicators for moderately increased albuminuria.

| **Variables** | **AUC** | **p value** | **Optimal cut-off value** | **Sensitivity** | **1 − Specificity** | **Youden index** |
| --- | --- | --- | --- | --- | --- | --- |
| TyH-i | 0.572 | < 0.001 | 6.669 | 0.404 | 0.256 | 0.148 |
| CHG index | 0.573 | < 0.001 | 5.865 | 0.503 | 0.363 | 0.140 |
| HbA1c | 0.577 | < 0.001 | 8.750 | 0.664 | 0.528 | 0.136 |
| FPG | 0.562 | < 0.001 | 8.305 | 0.579 | 0.447 | 0.132 |
| TYG index | 0.567 | < 0.001 | 9.860 | 0.260 | 0.146 | 0.114 |
| TyG-AIP | 0.558 | 0.001 | 5.788 | 0.304 | 0.202 | 0.102 |
| TYG/HDL-c | 0.555 | 0.002 | 9.460 | 0.392 | 0.281 | 0.111 |
| TG | 0.551 | 0.004 | 1.665 | 0.439 | 0.348 | 0.091 |

**Note**：ROC curve analysis is employed to diagnose the predictive efficacy of different indices.

**Abbreviations**: TyH-i, triglyceride-glycated hemoglobin index; CHG index, cholesterol-high-density lipoprotein-glucose index; HbA1c, glycosylated hemoglobin; FPG, fasting plasma glucose; TyG index, triglyceride–glucose index; TyG-AIP, triglyceride glucose (TyG)-atherogenic index of plasma (AIP) index; TyG/HDL-c, triglyceride-glucose index to high-density lipoprotein cholesterol ratio; TG, triglyceride.

**Appendix Table 4** | Pairwise comparison of ROC curves (DeLong test)

| **Item 1** | **Item 2** | **Difference in AUC** | **SE** | **95% CI** | **z *value*** | **P** |
| --- | --- | --- | --- | --- | --- | --- |
| TyH-i | CHG index | 0.0012 | 0.0142 | -0.027 - 0.029 | 0.0869 | 0.9307 |
| TyH-i | HbA1c | 0.0057 | 0.0175 | -0.029 - 0.040 | 0.3223 | 0.7472 |
| TyH-i | FPG | 0.0093 | 0.0179 | -0.026 - 0.044 | 0.5182 | 0.6043 |
| TyH-i | TyG index | 0.0044 | 0.008 | -0.011 - 0.020 | 0.5473 | 0.5842 |
| TyH-i | TyG-AIP | 0.0135 | 0.0079 | -0.002 - 0.029 | 1.7086 | 0.0875 |
| TyH-i | TyG/HDL-c | 0.0164 | 0.0164 | -0.016 - 0.049 | 1.0043 | 0.3153 |
| TyH-i | TG | 0.0209 | 0.0068 | 0.008 - 0.034 | 3.0796 | 0.0021 |
| CHG index | HbA1c | 0.0044 | 0.0184 | -0.032 - 0.040 | 0.2402 | 0.8101 |
| CHG index | FPG | 0.0105 | 0.0102 | -0.009 - 0.030 | 1.0331 | 0.3015 |
| CHG index | TyG index | 0.0056 | 0.0105 | -0.015 - 0.026 | 0.5354 | 0.5924 |
| CHG index | TyG-AIP | 0.0147 | 0.0144 | -0.014 - 0.043 | 1.0203 | 0.3076 |
| CHG index | TyG/HDL-c | 0.0177 | 0.0166 | -0.015 - 0.050 | 1.0678 | 0.2856 |
| CHG index | TG | 0.0221 | 0.0163 | -0.010 - 0.054 | 1.3543 | 0.1756 |
| HbA1c | FPG | 0.0149 | 0.0177 | -0.020 - 0.050 | 0.8417 | 0.3999 |
| HbA1c | TyG index | 0.01 | 0.02 | -0.029 - 0.049 | 0.5011 | 0.6163 |
| HbA1c | TyG-AIP | 0.0191 | 0.022 | -0.024 - 0.062 | 0.8705 | 0.384 |
| HbA1c | TyG/HDL-c | 0.0221 | 0.0227 | -0.022 - 0.067 | 0.9728 | 0.3307 |
| HbA1c | TG | 0.0265 | 0.0225 | -0.018 - 0.071 | 1.1783 | 0.2387 |
| FPG | TyG index | 0.0049 | 0.013 | -0.021 - 0.030 | 0.3768 | 0.7063 |
| FPG | TyG-AIP | 0.0042 | 0.019 | -0.033 - 0.041 | 0.2207 | 0.8253 |
| FPG | TyG/HDL-c | 0.0072 | 0.0207 | -0.033 - 0.048 | 0.3463 | 0.7291 |
| FPG | TG | 0.0116 | 0.02 | -0.028 - 0.051 | 0.5801 | 0.5619 |
| TyG index | TyG-AIP | 0.0091 | 0.0086 | -0.008 - 0.026 | 1.0518 | 0.2929 |
| TyG index | TyG/HDL-c | 0.0121 | 0.0164 | -0.020 - 0.044 | 0.7363 | 0.4615 |
| TyG index | TG | 0.0165 | 0.0087 | -0.001 - 0.034 | 1.9005 | 0.0574 |
| TyG-AIP | TyG/HDL-c | 0.003 | 0.0112 | -0.019 - 0.025 | 0.2654 | 0.7907 |
| TyG-AIP | TG | 0.0074 | 0.0056 | -0.004 - 0.018 | 1.3226 | 0.186 |
| TyG/HDL-c | TG | 0.0044 | 0.016 | -0.027 - 0.036 | 0.2784 | 0.7807 |

**Note**: Pairwise comparison of AUC values for each predictive index was conducted using DeLong’s test.

**Abbreviations**: TyH-i, triglyceride-glycated hemoglobin index; CHG index, cholesterol-high-density lipoprotein-glucose index; HbA1c, glycosylated hemoglobin; FPG, fasting plasma glucose; TyG index, triglyceride–glucose index; TyG-AIP, triglyceride glucose (TyG)-atherogenic index of plasma (AIP) index; TyG/HDL-c, triglyceride-glucose index to high-density lipoprotein cholesterol ratio; TG, triglyceride.

**Appendix Table 5 |** NRI after adding glucose-lipid indices to the baseline risk model.

| **Models** | **Variables** | **NRI** | **P** | **95%CI** |
| --- | --- | --- | --- | --- |
| Model 1 | HbA1c | 32.503% | <0.001 | 20.703% - 44.304% |
| Model 2 | TyH-i | 25.762% | <0.001 | 13.936% - 37.587% |
| Model 3 | CHG index | 22.429% | <0.001 | 10.593% - 34.264% |
| Model 4 | TyG index | 11.580% | 0.056 | -0.310% - 23.470% |
| Model 5 | FPG | 24.516% | <0.001 | 12.683% - 36.349% |
| Model 6 | TyG-AIP | 13.223% | 0.029 | 1.331% - 25.115% |
| Model 7 | TyG/HDL-c | 8.095% | 0.182 | -3.804% - 19.995% |
| Model 8 | TG | -5.010% | 0.408 | -16.870% - 6.851% |

**Note**: The baseline model was adjusted for conventional clinical covariates, including age, duration of diabetes, VFA, SBP, TC, HDL-c, FINS, GGT, UA, eGFR, HOMA-IR, Hb, ACEIs or ARBs, Metformin, Insulin, DR. NRI values are presented as percentages.

Model 1: Common confounding variables + HbA1c;

Model 2: Common confounding variables + TyH-i;

Model 3: Common confounding variables + CHG index;

Model 4: Common confounding variables + TyG index;

Model 5: Common confounding variables + FPG;

Model 6: Common confounding variables + TyG-AIP;

Model 7: Common confounding variables + TyG/HDL-c;

Model 8: Common confounding variables + TG.

**Appendix Table 6 |** IDI assessing incremental discrimination of glucose-lipid indices.

| **Models** | **Variables** | **IDI** | **P** | **95%CI** |
| --- | --- | --- | --- | --- |
| Model 1 | HbA1c | 0.035 | <0.001 | 0.023 - 0.047 |
| Model 2 | TyH-i | 0.015 | 0.006 | 0.004 - 0.025 |
| Model 3 | CHG index | 0.017 | 0.004 | 0.005 - 0.028 |
| Model 4 | TyG index | 0.009 | 0.086 | -0.001 - 0.019 |
| Model 5 | FPG | 0.017 | 0.002 | 0.007 - 0.028 |
| Model 6 | TyG-AIP | 0.010 | 0.012 | 0.002 - 0.017 |
| Model 7 | TyG/HDL-c | 0.003 | 0.533 | -0.007 - 0.014 |
| Model 8 | TG | -0.003 | 0.546 | -0.011 - 0.006 |

**Note**: The baseline model was adjusted for conventional clinical covariates, including age, duration of diabetes, VFA, SBP, TC, HDL-c, FINS, GGT, UA, eGFR, HOMA-IR, Hb, ACEIs or ARBs, Metformin, Insulin, DR. IDI values are presented as decimal values.

Model 1: Common confounding variables + HbA1c;

Model 2: Common confounding variables + TyH-i;

Model 3: Common confounding variables + CHG index;

Model 4: Common confounding variables + TyG index;

Model 5: Common confounding variables + FPG;

Model 6: Common confounding variables + TyG-AIP;

Model 7: Common confounding variables + TyG/HDL-c;

Model 8: Common confounding variables + TG.

**Appendix Figure 1** The heatmap displays the Spearman correlation coefficients among all variables.

**Note**: A univariate correlation analysis was conducted to identify the factors associated with moderately increased albuminuria. Blue colors show strong positive relationships (coefficients close to +1), red colors show strong negative relationships (coefficients close to −1), and near-white regions show weak or negligible relationships (coefficients close to 0).

**Abbreviations**: Moderately increased albuminuria: UACR 30-300mg/g; BMI, body mass index; VFA, visceral fat area; SFA, subcutaneous fat area; SBP, systolic blood pressure; DBP, diastolic blood pressure; TC, total cholesterol; TG, triglyceride; HDL-c, high-density lipoprotein cholesterol; LDL-c, low-density lipoprotein cholesterol; FPG, fasting plasma glucose; FINS, fasting serum insulin; HbA1c, glycosylated hemoglobin; HOMA-IR, homeostatic model assessment of insulin resistance; AST, aspartate aminotransferase; ALT, alanine aminotransferase; GGT, gamma-glutamyl transferase; UA, uric acid; eGFR, estimated glomerular filtration rate;

Hb, hemoglobin; SGLT2 inhibitors, Sodium glucose co-transporter 2 inhibitors; GLP-1 receptor agonists, glucagon-like peptide-1 receptor agonists; ACEI, angiotensin-converting enzyme inhibitor; ARB, angiotensin II receptor blocker; TyG index, triglyceride–glucose index; TYG/HDL-c, triglyceride-glucose index to high-density lipoprotein cholesterol ratio; TyG-AIP, triglyceride glucose (TyG)-atherogenic index of plasma (AIP) index; CHG index, cholesterol-high-density lipoprotein-glucose index; TyH-i, triglyceride-glycated hemoglobin index; DR, diabetic retinopathy.

**Appendix Figure 2 |** (A) Lasso regression coefficient trajectory plot; (B) 10-Fold cross-validation curve.

**Note:** LASSO regression with 10-fold cross-validation was performed to screen potential variables associated with moderately increased albuminuria in the entire population. The optimal λ was selected using lambda.1se.

**Appendix Figure 3 |** ROC analyses for moderately increased albuminuria.

Note: ROC, receiver‑operating characteristic; Moderately increased albuminuria, UACR 30-300mg/g; AUC, area under the curve.
